# Pharmacokinetics of Non-β-Lactam β-Lactamase Inhibitors

**DOI:** 10.3390/antibiotics10070769

**Published:** 2021-06-24

**Authors:** Giacomo Luci, Francesca Mattioli, Marco Falcone, Antonello Di Paolo

**Affiliations:** 1 Department of Clinical and Experimental Medicine, University of Pisa, Via Savi 10, 56126 Pisa, Italy; giacomo.luci@for.unipi.it (G.L.); marco.falcone@unipi.it (M.F.); 2 Department of Internal Medicine, Pharmacology & Toxicology Unit, University of Genoa, 16100 Genoa, Italy; fmattiol@unige.it

**Keywords:** avibactam, vaborbactam, relebactam, durlobactam, pharmacokinetics, pharmacokinetics/pharmacodynamics

## Abstract

The growing emergence of drug-resistant bacterial strains is an issue to treat severe infections, and many efforts have identified new pharmacological agents. The inhibitors of β-lactamases (BLI) have gained a prominent role in the safeguard of beta-lactams. In the last years, new β-lactam–BLI combinations have been registered or are still under clinical evaluation, demonstrating their effectiveness to treat complicated infections. It is also noteworthy that the pharmacokinetics of BLIs partly matches that of β-lactams companions, meaning that some clinical situations, as well as renal impairment and renal replacement therapies, may alter the disposition of both drugs. Common pharmacokinetic characteristics, linear pharmacokinetics across a wide range of doses, and known pharmacokinetic/pharmacodynamic parameters may guide modifications of dosing regimens for both β-lactams and BLIs. However, comorbidities (i.e., burns, diabetes, cancer) and severe changes in individual pathological conditions (i.e., acute renal impairment, sepsis) could make dose adaptation difficult, because the impact of those factors on BLI pharmacokinetics is partly known. Therapeutic drug monitoring protocols may overcome those issues and offer strategies to personalize drug doses in the intensive care setting. Further prospective clinical trials are warranted to improve the use of BLIs and their β-lactam companions in severe and complicated infections.

## 1. Introduction

For several decades, the surge of resistant bacterial strains and the severity of their infections have represented the most compelling emergences in antimicrobial chemotherapy. Indeed, the efficacy of drugs may last for a limited time due to the progressive selection of resistant strains by several causes (i.e., excessive prescription and misuse of drugs). Among β-lactams, what happened to methicillin is still a paradigmatic example of drug inactivation by β-lactamase (BL) enzymes. Overall, BLs can inactivate β-lactams and have a pivotal role in treatment failures, reduction of therapeutic options, and the emergence of resistance [1,2]. Therefore, preclinical research and many clinical trials are evaluating new bactericidal β-lactams and β-lactamase inhibitors (BLI).

Different clones of the same bacterial species may display unrelated expression patterns of BLs inactivating enzymes; thus, some authors have postulated that the dose of BLI would be based on the turnover of the BLs to bring the strain susceptibility below the established breakpoints for the β-lactams alone [3]. However, it could be difficult to optimize the dose of BLIs based on BL expression, because the latter is not always associated with the antibacterial efficacy of the β-lactam [4].

It is worth noting that the PK/PD characteristics of BLIs resemble that of their β-lactam companions. It is widely accepted that prolonged or continuous intravenous (IV) infusions of β-lactams may maximally exploit their bactericidal effect owing to their time-dependent killing [5,6]. Therefore, the bactericidal effect is better predicted by the percentage of time between two consecutive administrations during which plasma concentrations are higher than MIC value (%T > MIC). Several studies have demonstrated that for some BLIs a threshold concentration (C_t_) higher than a critical value or a free-drug area under the curve (*f*AUC) over MIC ratio (*f*AUC/MIC) could represent the PK/PD parameters that predict the BLI efficacy and may guide the choice of the most appropriate dosing regimen. In other words, the efficacy of a β-lactam–BLI combination may depend on the relationships among the pharmacokinetics of the BLI, its dosing regimen, and the type/expression of BLs against which the BLI has a variable substrate affinity [7]. Consequently, BLI threshold values are “*isolate/enzyme dependent*” [8].

The possibilities of optimizing chemotherapeutic regimens may be limited and rapid changes in patient’s clinical conditions may require frequent dose adjustments. However, β-lactams and BLIs have similar PK characteristics that may help treatment optimization. Indeed, the hydrophilic structure of both β-lactams and BLIs limits absorption and tissue distribution, while renal excretion represents the main excretory pathway. Consequently, those factors that may alter drug disposition, as well as “third space”, renal impairment and renal replacement therapies (RRT), affect both β-lactams and BLIs [9]. Due to common pharmacokinetic characteristics (i.e., similar plasma half-lives) shared by β-lactams and BLIs, dose optimization of BLI may mirror the changes in dosing regimens of β-lactams. Finally, the activity spectrum of some β-lactam–BLI combinations may be effective in difficult-to-treat infections, owing to a synergic interaction between the two drugs. For example, aztreonam (ATM), in combination with ceftazidime (CAZ)–avibactam (AVI), is effective against Gram-negative *Enterobacterales*, producing metallo-BLs (MBLs) [10].

The following paragraphs will offer a timely update vision of BLIs to the readers, with a special reference to the pharmacokinetics of drugs, those factors responsible for individual variability, and the pharmacokinetic/pharmacodynamic characteristics (PK/PD).

## 2. Structure and Mechanism of Action

**Figure 1:**
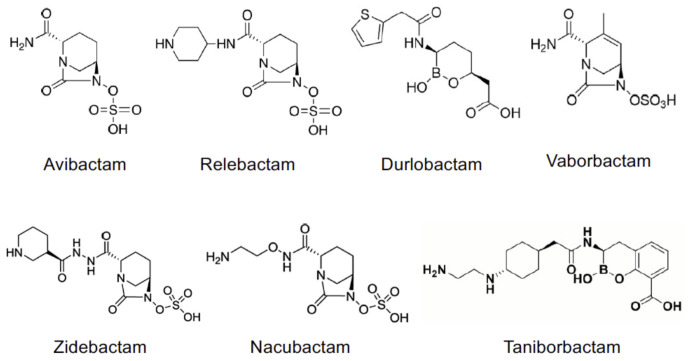
Chemical structures of non-beta-lactam BLIs.

In origin, the former molecules as clavulanic acid, sulbactam, and tazobactam, share the same chemical core as penicillin (Figure 1).

In particular, sulbactam {SUL, (2S,5R)-3,3-dimethyl-4,4,7-trioxo-4λ^6^-thia-1-azabicyclo[3.2.0]heptane-2-carboxylic acid} and its congener tazobactam {TAZ, (2S,3S,5R)-3-methyl-4,4,7-trioxo-3-(1H-1,2,3-triazol-1-ylmethyl)-4λ^6^-thia-1-azabicyclo[3.2.0]heptane-2-carboxylic acid} are penicillanic acid sulfones that act as suicide molecules and irreversible inhibitors of Ambler class A serine BLs (see below). Through the formation of intermediate complexes, these BLIs covalently bind their target BLs. The bond formation is slow but irreversible, with the complete and definitive inhibition of the enzyme. However, these BLIs may undergo hydrolysis catalyzed by the BL; thus, the efficiency of target inhibition depends on the formation rate of the enzyme-BLI inactive complex rather than the inactivation of the BLI. Consequently, the number of BLI molecules that are required to inhibit the same BL may differ [11].

The most recent BLIs belong to diazabicyclo[3.2.1]octanone (DBO), boronic acid and pyridine-2-carboxylic acid classes, and they offer a different binding kinetics with respect to the oldest penicillanic acid sulfones. Avibactam {AVI, [(2*S*,5*R*)-7-oxo-1,6-diazabicyclo[3.2.1]octane-2-carboxamide]}, relebactam {REL, (1*R*,2*S*,5*R*)7-oxo-2-(piperidin-1-ium-4-ylcarbamoyl)-1,6-diazabicyclo[3.2.1]octan-6-yl sulfate} and durlobactam {DUR, [(2*S*,5*R*)-2-carbamoyl-3-methyl-7-oxo-1,6-diazabicyclo[3.2.1]oct-3-en-6-yl] hydrogen sulfate} commonly show the presence of a DBO moiety, as well as zidebactam {ZID, [(1*R*,2*S*,5*R*)-7-oxo-2-({[(3*R*)-piperidin-3-yl]formohydrazido}carbonyl)-1,6-diazabicyclo[3.2.1]octan-6-yl]oxidanesulfonic acid} and nacubactam {NAC, [(1*R*,2*S*,5*R*)-2-[(2-aminoethoxy)carbamoyl]-7-oxo-1,6-diazabicyclo[3.2.1]octan-6-yl]oxidanesulfonic acid}. Vaborbactam {VAB, (3*R*,6*S*)-2-hydroxy-3-[[2-(2-thienyl)acetyl]amino]-1,2-oxaborinane-6acetic acid} and taniborbactam {TAN, (3*R*)-2-hydroxy-3-{2-[(1*r*,4*r*)-4-[(2-aminoethyl)amino]cyclohexyl]acetamido}-3,4-dihydro-2*H*-1,2-benzoxaborinine-8-carboxylic acid} are characterized by the presence of a cyclic boronic acidic scaffold. The inhibitory activity of new BLIs is broad and more potent than that of β-lactam BLIs, as it was formerly demonstrated for AVI [12]. Moreover, DBO compounds are capable of inhibiting penicillin-binding proteins (PBP) thus showing a “β-lactam enhancer” activity [13,14] and a synergistic bactericidal activity in combination with β-lactam [15], even against MBL-producing bacteria [16,17].

The non-β-lactam structure confers innovative characteristics. As a matter of fact, these drugs may resist BL hydrolysis to some extent and can bind the target in a rapid and reversible manner, while the regenerated BLI may interact with its target several times, resulting in an efficient and long-lasting inhibition.

AVI acylates the BL and its cyclic urea ring opens, but the BLI can recyclize and dissociate in an intact form. The dissociation rate may depend on the specific enzyme, being widely variable for AmpC BLs (7–1600 nM) and showing the highest value for FOX-4 BL [18]. However, the dissociation can end with the inactivation of the BLI, as it occurs when AVI binds KPC-2. REL shares the same core structure of AVI, the mechanism of action is identical, and the BLI-enzyme complex is stable and long lasting [19].

DUR recyclizes and dissociates intact from Ambler class A and C BLs, as well as AmpC, CTX-M-15, P99, SHV-5, and TEM-1, but not from other classes A and D BLs, such as KPC-2, OXA-10, OXA-23, OXA-24, or OXA-48 [20]. The recycling of BLI from the BL also depends on the inactivation rate of the drug, as measured by the variable partition ratio value, which is inversely correlated with the recycling rate. For instance, DUR has a partition ratio close to 1 for many BLs, but that ratio increases to 3.0 after 2 h of exposure to KPC-2 [20]. In the case of ZID, the BLI is more potent in the reversible acylation of AmpC, while the recycling from CTX-M-15 is faster than AVI and REL [15].

According to its activity as a competitive inhibitor, VAB covalently binds class A and C BLs in a two-step reaction [21]. The dissociation rate of VAB differs widely among the different BLs (from 50 up to 200 folds), showing a fast off rate for SHV-12 and TEM-43 (likely due to an unstable covalent bond), and a low off rate for KPC. Those values explain why VAB may increase the antibacterial activity of drugs against KPC-producing strains rather than against SHV or TEM.

Crystallographic studies have demonstrated that TAN interacts with class A, C, and D BLs in the closed or cyclic boronate form [22], mimicking the tetrahedral anionic intermediate in serine BLs >[23]. More interestingly, the boronate-based BLI (made by a bicyclic boronate fused to a benzoic acid) may also inhibit different MBLs, making TAN a pan-inhibitor of BLs, as explained below (Table 1).

## 3. Spectrum of Activity of BLIs and Mechanisms of Resistance Structure and Mechanism of Action

The spectrum of activity may differ among BLIs. Indeed, as “first-generation” molecules, SUL and TAZ are more potent than CLA against Ambler class C cephalosporinases (AmpC) and class A carbapenemases (KPC) [24]. SUL has inhibitory action against plasmid-mediated BLs, while TAZ is more potent than SUL against TEM enzymes, while both SUL and TAZ are not effective against MBLs. The most recent BLIs have a large spectrum of activity against several BLs, including Ambler class C, D, and B BLs (Table 1).

AVI can inhibit Ambler class A and C BLs [30], also possessing a weak intrinsic antibacterial activity [31]. However, AVI does not inactivate class B MBL and many oxacillinases (OXA, Ambler class D) [32]. REL has a spectrum of activity matching that of AVI against class A (KPCs) and class C BLs [33], but it lacks any effect against OXA BLs produced by *Acinetobacter baumannii* and class B MBL (e.g., NDM, IMP, VIM) [33,34].

VAB specifically counteracts the *K. pneumoniae* carbapenemase (KPC) [35,36] and its spectrum of activity includes many class A and class C BLs, as the newly identified class A carbapenemases BKC-1 and FRI-1 found in *K. pneumoniae* and *Enterobacter cloacae* isolates, respectively [34]. VAB is ineffective against class B MBL and class D BLs.

DUR is an expanded-spectrum BLI that potently inhibits clinically relevant class A, C, and D BL, resulting in intrinsic antibacterial activity against *Enterobacteriaceae*, and it regenerates β-lactam activity in a broad range of MDR Gram-negative pathogens [37]. In comparison with AVI, DUR showed a better potentiation of the antibacterial activity of piperacillin against classes A, C, and D BLs, and, in combination with imipenem, it was superior to AVI against OXA BLs [20].

In agreement with DUR, ZID rapidly acylates class A, C and D BLs including CTX-M-like, AmpC, and OXA-48 BLs [27,28], and it also has potent inhibitory activity against *Pseudomonas aeruginosa* and *A. baumannii* PBP-2 [15,38]. Notably, the inhibition of PBP-2 restored the sensitivity to ZID plus cefepime in *P. aeruginosa* strains producing class B MBL, even though ZID did not inhibit VIM-2 [38]. Similar results were observed in *Enterobacteriaceae* producing MBL when exposed to NAC in combination with meropenem or cefepime [39]. In agreement with those findings, NAC is active against serine BLs, such as TEM, AmpC, SHV, CTX-M and OXA BLs, and the MBL NDM [17,40]. Moreover, the drug is a PBP-2 inhibitor, thus proving effective in combination with meropenem against *Enterobacterales* resistant to the carbapenem.

Finally, TAN is a pan-inhibitor of BLs because its spectrum extends to both serine- and metallo-BLs, embracing all four Ambler classes [22]. Its inhibitory activity against class A, C, and D BLs is comparable with that of AVI, but its IC_50_ values against NDM-1 and VIM-2 MBLs are in the range 0.03–0.1 µM, whereas AVI and VAB values are >100 µM.

Several mechanisms may have a role in chemoresistance. The overexpression of BLs (caused by increased gene copy number) or their mutational status confer a vital advantage to bacteria strains [41,42,43]. The reduced sensitivity to the β-lactam–BLI combination may also depend on the reduced expression of porins on the outer membrane of Gram-negative bacteria and the presence of membrane efflux pumps. For example, the genetic resistance to CAZ-AVI may depend on both *KPC-2* gene amplification and mutation of porin coding genes that encode (a) nonfunctional Ompk35 or (b) Ompk36 with low activity [41,44]. Therefore, there is “*the need to optimize the use of current agents to minimize the emergence of resistance*” [41].

## 4. Pharmacokinetics of BLIs

BLIs have a hydrophilic structure that may influence their pharmacokinetics as that of β-lactam companions. Except for CLA, BLIs have a low oral bioavailability that requires an IV administration, a volume of distribution (Vd) limited to the extracellular space, and reduced plasma protein binding. Overall, the maximum interindividual variability in drug pharmacokinetics accounts for 35%. The relative hydrophilicity allows the renal excretion of BLIs with a negligible hepatic metabolism so that the change in renal function is the first cause of dose adjustment. Among the non-β-lactam BLIs, AVI is a reference because of its larger mass of data with respect to other molecules (Table 2).

### 4.1. Linear Pharmacokinetics

One main characteristic is the linear pharmacokinetics of BLIs that allows prompt dose adjustments when they could be required. For instance, AVI displays linear pharmacokinetics after single 30 min IV infusions (dose range, 50 mg–2 g) in healthy male volunteers (HV), with little or no drug accumulation after multiple IV infusions (0.5–1 g q8h for up to 10 days) [58]. The systemic clearance of DUR did not change after single (0.25–8.0 g) and multiple doses (0.25–2.0 g) [59]. Similar findings were obtained for VAB (dose range, 0.25–2.0 g) [60] and REL (dose range 0.025–1.15 g) [61], while NAC has linear pharmacokinetics in HV when administered as single (50–8000 mg) or multiple doses (1–4 g q8h for up to 7 days) [55]. Those findings demonstrated that multiple doses were not associated with drug accumulation [54,62], as demonstrated for TAN [57]. The linear pharmacokinetics of REL, VAB, DUR, and NAC is not affected by the coadministration of β-lactam companions (i.e., imipenem/cilastatin, meropenem, sulbactam) [34,55,59,61]. Even after multiple doses, AVI maintains its linear pharmacokinetics in combination with CAZ [62].

It is worth noting that the dose range characterized by linear pharmacokinetics includes the doses that have been registered or are under clinical evaluation, hence reinforcing the possibility of dose adjustments.

### 4.2. Distribution

The estimated V_d_ at steady state (V_d,ss_) of BLIs is approximately 18–25 L, and population pharmacokinetic studies describe it by two-compartment models [34,58,59,60,63]. It is of note that body weight may influence the distribution of BLI. For example, subjects at the 10th (51 kg) or 90th percentile (95 kg) of body weight distribution had an estimated volume of the central compartment (V_c_) 29% lower or 39% higher than patients at the median weight (70 kg), respectively [62].

The V_d_ of BLIs is limited to the interstitial space because of the hydrophilic properties of the molecules, but BLIs may achieve relatively high concentrations in some tissues. AVI diffuses into the human bronchial epithelial lining fluid (ELF) with concentrations (in terms of area under the time–concentration curve, (AUC)) around 30% of those in plasma, and concentration–time profiles are similar in ELF and plasma [32,64,65]. In a study enrolling HV, the ELF/plasma penetration ratio was 0.42, with ELF concentrations (1.4 mg/L) higher than the corresponding PK/PD target values (1 mg/L) 4 h after dosing (i.e., the midpoint of the 8 h dosing interval) [65]. In cystic fibrosis patients, serum pharmacokinetics of AVI reflects that obtained in HV [66], with mean maximum concentration (C_max_) values in sputum of 1.53 mg/L 2 h after administration, and maximum and overall sputum/plasma penetration ratios of 0.1 and 0.13, respectively.

Data collected from 5 phase II clinical trials helped to increase our knowledge about AVI pharmacokinetics in the presence of severe infections [62]. V_c_ values in patients with complicated intraabdominal infections (cIAI) and complicated urinary tract infections (cUTI) were, respectively, 32.9% and 43.4% higher than those measured in HV. However, the difference in the steady-state pharmacokinetics of AVI between several subgroups of patients and HV was lower than 20%. Critically ill patients with several comorbidities (i.e., cancer, diabetes, etc.) and burns had larger V_d_ of AVI (median, 40.2 L) [67], in agreement with a previous study (mean V_d_, 50.8 L) [68].

After the administration of VAB 2 g plus MER 2 g in HV, VAB had an ELF diffusion greater than AVI, with mean ELF/plasma AUC_0–8h_ ratios of 0.79 for unbound plasma concentrations [69]. Interestingly, in alveolar macrophages, MER was not detectable, whereas VAB achieved concentrations in the range 2.35–6.94 mg/L. For DUR, the median ELF/plasma AUC_0–6h_ ratio values were 0.41 and 0.40, taking into consideration the total and unbound plasma concentrations, respectively [70]. The patient’s body weight affected the V_c_ of REL and imipenem [71]. Multiple doses of REL 0.25 g achieved an ELF/plasma AUC ratio of 0.54 for unbound plasma concentrations, while that ratio was 0.36 in alveolar cells [72]. In HV, multiple doses of ZID 1 g plus cefepime 2 g q8h were associated with a mean ELF/plasma AUC_0–8h_ ratio of ZID equal to 0.39 (range 0.31–0.95) [54]. The mean penetration of ZID in alveolar macrophages accounted for 10% of plasma concentrations up to 8 h post-dose.

Data regarding the distribution of BLIs in tissues other than those of the respiratory tract are scarce. The blood–brain barrier represents an obstacle to the liquor diffusion of AVI, as demonstrated by the higher liquor/plasma AUC ratio with inflamed meninges (0.38) [45], a pathological condition that may affect the penetration of many antimicrobial drugs into the central nervous system [73].

Finally, the plasma protein binding is variable, being lowest for AVI (8%) and highest for VAB (33%) [72,74], and it marginally contributes to the disposition of BLIs. However, differences in plasma protein binding could be responsible for pharmacokinetic variations between BLIs and β-lactam companions during RRT [75].

### 4.3. Biotransformation

Owing to their hydrophilic structure, BLIs are primarily excreted intact through the kidneys with a minimal liver metabolism [35]. For example, in vitro experiments using human liver tissue preparations (microsomes and hepatocytes) demonstrated that AVI biotransformation is negligible, while its excretion within the feces is approximately 0.25% of a dose [76]. Notably, the decreased hepatic metabolism of BLIs reduces the risk for clinically relevant drug–drug interactions (DDIs). AVI did not show significant inhibition/induction of cytochrome P450 enzymes in vitro. Using microsome preparations or freshly isolated hepatocytes from donors, AVI weakly inhibits CYP2C9 and induces CYP2E1 only at 5 mM (approximately 1.3 g/L) [77], a concentration that is unlikely achievable in tissues after the administration of standard doses. Furthermore, liver parenchyma does not metabolize VAB; therefore, dose adjustments are not required in the presence of the liver impairment [78]. Finally, a clinical study in HV demonstrated a negligible metabolism of NAC [55].

### 4.4. Excretion

The renal excretion of BLIs is relatively rapid, as demonstrated by the short terminal half-lives (t_1/2_) of these drugs ranging from 0.5 up to 2.2 h (Table 2). Approximately 97% of the AVI dose is recovered in urine, 95% within 12 h following multiple doses [24,58,63]. The fraction of REL excreted in urine did account for 94.7–100% in the first 24 h after a single dose in HV [61]. The urinary excretion of VAB accounts for 81–99% over 48 h [79,80], whereas it represents only 66% of the systemic clearance (CL) of DUR [81]. The percentage of an administered dose that is excreted through the kidney accounts for nearly 100% and 88.4% for ZID and NAC, respectively [54,55].

Overall, the calculation of CL returns dose-independent values of approximately 9–10 L/h [82]. The creatinine clearance (CrCL) is the main covariate predicting the CL [62]. For example, the mean AVI AUC increased 3.8 and 7 times in subjects with moderate and severe renal impairment, respectively. Therefore, dose adjustments are mandatory for patients with CrCL <50 mL/min because they are associated with an appreciable reduction of AVI CL. In critically ill patients, an APACHE II score >10 (a greater disease severity according to an integrated scoring system) was associated with a decrease in drug CL of 19.7%, and non-Chinese, non-Japanese Asian patients had an 8.65% lower CL than the other individuals [46]. Finally, the mean predicted value of AVI CL in cystic fibrosis patients (12.3 L/h) did match that obtained in HV [66].

Changes in dosing regimen are not necessary for patients with augmented renal clearance (ARC) [32,62], which may alter the pharmacokinetics of drugs that are mainly excreted through the kidneys [82]. Indeed, the effect of ARC on AVI pharmacokinetics was considered modest (factor, 0.992), dose adjustment not warranted, and PK/PD targets achievable because the analysis predicted an effective treatment in more than 90% of patients regardless of the infection, such as cIAI, cUTI, pyelonephritis, nosocomial pneumonia including hospital-acquired pneumonia and ventilator-associated pneumonia [62].

The exposure to VAB linearly increased with the progressive reduction of estimated glomerular filtration rate (eGFR) after the administration of a 1 g single dose [79]. Population pharmacokinetic analyses showed that the exposure to REL increased more than three times in patients with severe renal impairment (CrCl, 15–<30 mL/min) [71]. Furthermore, changes in REL exposure occurred in older people and adult women populations [61]. In patients with renal impairment who received halved doses, DUR exposure doubled in the presence of CrCL values <30 mL/min/1.73 m^2^ [81].

Changes in ZID pharmacokinetics were significantly associated with renal impairment [83]. Indeed, halved doses of ZID and cefepime (respectively, 0.5 plus 1 g q8h) resulted in systemic exposure (in terms of AUC_0–∞_ values) higher than matched HV, with geometric mean ratios of 2.9 and 9.1 in severe renal impairment and in ESRD on HD, respectively. The terminal t_1/2_ increased up to 12.9 h in severe renal impairment. Finally, clearance changes in ZID were superimposable with those calculated for cefepime within the range of CrCL values. Even in the case of TAN, severe renal impairment was associated with a 4.5-fold decrease in CL, which matched a similar reduction of cefepime CL [56].

It is worth noting that the pharmacokinetics of BLIs in patients with severely compromised renal function may be altered by the presence of hemodialysis (HD) that can remove nearly half a dose of AVI and VAB [79,84,85,86]. In particular, the mean increase in systemic CL of VAB was 5.11-fold during HD [79], while median predicted values of HD CL for VAB and MER did not significantly differ (7.9 and 5.68 L/h, respectively) [87]. ESRD patients receiving DUR showed similar findings [81].

The increased excretion by the dialytic techniques may partly counteract the extreme reduction of BLI CL in severe renal impairment so that dose adjustments are needed. For example, in ESRD patients on HD, doses of imipenem-REL 0.2/0.1 g q6h administered after HD were associated with the achievement of target PK/PD targets [71], hence recommending the administration of the BLIs after intermittent HD [78]. Scarce data are available for the most recent techniques of continuous renal replacement therapy (CRRT), as well as continuous venovenous HD (CVVHD) or hemodiafiltration (CVVHDF) [88]. In a patient, the CL of AVI due to CVVHD was 54.3% of the total BLI CL (2.95 L/h) [85], while the value of systemic AVI CL was 1.45 L/h during CVVHDF [89]. In both cases, the β-lactam had CL changes that matched those of AVI. Those findings partly agreed with an ex vivo study, in which continuous venovenous hemofiltration (CVVH) efficiently cleared both VAB and MER, even if VAR CL was 20–40% lower than that of MER [90]. Interestingly, in three out of four patients with infections caused by pan-drug or extremely drug-resistant strains of *P. aeruginosa*, the multivariate analysis identified a significant correlation between CRRT and the failure of standard CAZ-AVI regimen [88].

Finally, it is worth noting that renal CL values also suggest an active tubular secretion of BLIs [24], which may have a variable affinity for transmembrane transporters. As a matter of fact, in vitro studies demonstrated that AVI is a substrate of OAT1 and OAT3 transporters, but the risk of DDI could be low [76]. AVI is not a substrate of ABCB1, ABCG2, and ABCC4 transporters [76], while further in vitro experiments confirmed that REL was not a substrate of transmembrane transporters of OAT, MATE, and OATP families with a low risk of clinically relevant DDIs [91].

## 5. Pharmacokinetic/Pharmacodynamic Relationships

In general, the pharmacokinetic/pharmacodynamic (PK/PD) characteristics of β-lactams are mainly associated with a time-dependent killing, through which the total daily dose is fractionated in three to four extended infusions to prolong the time during which bacteria are exposed to bactericidal concentrations. The PK/PD parameter that predicts the antibacterial effect is the percentage of the time interval between two consecutive doses during which the free drug concentration is above the pathogen MIC (%fT > MIC) [92]. The emergence of less sensitive or frankly resistant bacterial strains has brought to the identification of higher %*f*T > MIC values, with more aggressive dosing regimens that may guarantee C_min_ values four times the MIC (C_min_ > 4 × MIC), as in the case of meropenem [93]. That change partly reflects the negligible postantibiotic effect (PAE) and the post-BL inhibitor effect (PLIE) of β-lactams and BLIs, respectively, as observed for CAZ-AVI, ceftaroline-AVI, and ATM-AVI against bacterial strains producing different BLs, except CAZ-AVI against KPC-2-producing *Klebsiella pneumoniae* [94].

The best PK/PD index for BLIs is the percentage of time above a threshold concentration (C_t_) over the dosing interval (%*f*T > C_t_) [45], where the C_t_ value represents the minimum concentration of the BLI that ensures the inhibition of the BL and restores the antibacterial activity of the β-lactam [4]. In particular, the thigh and lung infection mouse models clearly showed that the AVI %*f*T > C_t_ threshold of 1 mg/L predicted bacterial stasis and 1 log kill against *P. aeruginosa* strains [4]. The threshold value did not change across the different evaluated regimens (i.e., AVI doses every 2 or 8 h) even if the more frequent administration (i.e., every 2 h) was associated with efficacy with a reduced daily dose. The PK/PD target value of 50% was adopted for AVI (*f*C_t_ > 1 mg/L) and CAZ (*f*C_t_ > 8 mg/L) [65,66].

The PK/PD parameter is dependent on many factors, among which one might include the variable expression of BLs, their affinity for BLIs, and the recycling rate of the enzyme-BLI complex so that in vitro and in vivo experiments allow the identification of the most appropriate C_t_ value [95]. For instance, the variable recycling rate of the AVI-enzyme complex depends on the residence time and half-life for deacylation, being longest for *E. cloacae* AmpC (300 min) and shortest against *P. aeruginosa* AmpC (6 min) [96]. On the contrary, some studies did not find any association between the MIC of β-lactams, C_t_ value of the BLI, and BL expression [4].

For other BLIs, the best predictive PK/PD parameter is the *f*AUC/MIC ratio, in which the denominator represents the MIC of BLI or even the MIC of the combined β-lactam in the presence of the BLI. An in vitro 2 log kill for REL was obtained when the *f*AUC/MIC ratio was ≥7.5 [97]. When combined with imipenem (with a *f*T > MIC ≥ 6.5), the REL target was a *f*AUC_0–24h_/MIC ratio ≥5.2 [71]. Again, in vivo experiments demonstrated that VAB efficacy was predicted by the *f*AUC_0–24h_/MIC ratio, in which the MIC value refers to MER in the presence of VAB at concentrations of 8 mg/L [98].

The most predictive PK/PD parameter (and its value) associated with the capability of the BLI in restoring the antimicrobial activity of β-lactams depends on both the preclinical models (i.e., the in vitro hollow fiber, the in vivo neutropenic mouse) and the β-lactam. Indeed, several studies evaluating AVI combined with CAZ, ceftaroline fosamil, and ATM showed that the best PK/PD index could be C_T_, *f*T > C_T,_ or *f*AUC [92]. Moreover, the *f*T > C_T_ threshold of AVI varied from 1 to 2.5 mg/L when the BLI was associated with CAZ and ATM, respectively.

Whatever the most predictive PK/PD parameter of BLI could be, the duration of exposure to concentrations above a threshold is a feature in common with β-lactams. That characteristic could increase the possibilities to achieve better clinical outcomes, even if some authors consider the availability of fixed-dose combinations an obstacle to dose optimization [24].

## 6. Discussion

The most recent BLIs represent appropriate options for the therapy of severe and difficult-to-treat infections caused by multiresistant bacterial strains. However, both pharmacodynamics and pharmacokinetics could be highly variable among patients depending on the expression of BLs and the individual’s clinical conditions.

The presence of multiple enzymes, their abundance, and mutational status certainly influence the outcome of chemotherapy [43], and appropriate preclinical models may evaluate PK/PD characteristics of BLIs in the presence of the β-lactam [92]. The time above a threshold concentration (%fT > Ct) and the *f*AUC over C_t_ or MIC values confer a time dependency to the activity of BLIs in restoring the efficacy of β-lactams against resistant strains. Therefore, every factor that may alter the pharmacokinetics of BLIs could reduce the attainment of desired PK/PD targets. In other words, the pharmacokinetic variability of drugs depends on the physical and chemical properties of BLIs in association with both clinical conditions of the patients and eventual additional medical interventions (i.e., HD, CVVHD, etc.). The pharmacokinetic studies demonstrated that CrCl is capable of significantly influencing the CL of BLIs, even if other concomitant factors (i.e., obesity, comorbidities, age, race) may contribute to the alteration of drug pharmacokinetics [61,67,88].

Therefore, the knowledge about the disposition and excretion of BLIs guides the discussion of some key points. Pharmacokinetic and pharmacometrics studies demonstrate that CrCl significantly affects the renal excretion of the BLI, and that relationship is linear or is approaching linearity [79,81]. Furthermore, a threshold value of GFR (40 or 50 mL/min) represents a pragmatic index to adjust the dosing regimen β-lactams–BLI combinations [45,50]. As a matter of fact, changes in renal excretion of BLIs (also including the intervention of the HD) may mirror those affecting the pharmacokinetics of β-lactam companions. For example, REL and imipenem changes according to renal impairment had the same magnitude (1.38–3.05-fold and 1.22–2.01-fold, respectively) [71], while the t_1/2_ values of both CAZ and AVI (2.3 and 2.2 h, respectively) increased to the same extent (5.17 and 5.92 h, respectively) in a patient with acute renal failure receiving CVVHDF [89]. Furthermore, β-lactams and their BLIs may also feature alterations in V_d_ [62,99]. Therefore, the dose adjustment can simultaneously involve both β-lactam and BLI, guaranteeing a dose modification of the same extent for both drugs across a wide interval of doses due to linear pharmacokinetics [67]. Some peculiar characteristics (for example, different plasma protein binding of drugs) could limit that approach.

The dosing regimen (Table 3) may be adjusted according to the severity of renal impairment, but comorbidities may contribute to utmost pharmacokinetic alterations that decrease the probability of PK/PD target attainment. In comorbid patients with severe renal impairment (i.e., eGFR, 15–30 mL/min), the registered dosing regimen is CAZ-AVI 0.75/0.1875 g q12h. However, high doses (i.e., CAZ-AVI 1/0.25 g q12h) may achieve a 90% probability of target attainment when CAZ MIC = 1 mg/L in the presence of AVI [67].

The relationship between renal impairment, comorbidities, and achievement of PK/PD targets is partially predictable a priori, such that a fine-tuning optimization of dosing regimen may be pursued by therapeutic drug monitoring (TDM) [105]. Chromatographic techniques are widely available to measure plasma concentrations of drugs [106,107,108], with turn-around time values that allow rapid changes in dosing regimen even in intensive care settings [109]. Furthermore, blood withdrawal at prespecified time points, as well as immediately before the next dose (to obtain the C_min_ value) or at midpoints (to evaluate whether the plasma concentration does exceed the target C_t_ value), can allow a quick check of predicted clinical outcome. In addition to this, population pharmacokinetic models may anticipate treatment efficacy in every patient, simulate different dosing regimens, and calculate PK/PD parameters (i.e., *f*AUC) from a sparse blood sampling scheme or based on TDM protocols [110,111].

In conclusion, the non-β-lactam BLIs available, in combination with β-lactam companions, represent effective therapeutic options to treat severe infections caused by BL-producing strains (Table 3). In the presence of renal impairment, the main factor influencing BLI disposition, the linear pharmacokinetics of these agents allow dose adjustments. However, other variables are possible causes of the large interindividual variability among critically ill patients; hence, future clinical studies will increase the knowledge about pharmacokinetics and PK/PD of β-lactam–BLI combinations. Finally, TDM protocols, modelling and simulation may help the timely optimization of dosing regimens, bringing personalized medicine in intensive care setting, and reducing the risk of resistance emergence.

## Figures and Tables

**Table 1:** Classification of BL and spectrum of activity of BLIs [2,17,22,24,25,26,27,28,29].

β-Lactamases			Substrates					Spectrum of Activity of BLIs						
Active Site	Ambler Class	Representative Enzymes												
			Pen	Cep	ECep	Cbn	Mb	AVI	REL	VAB	DUR	ZID	NAC	TAN
Serine	A	PC1	✓					✓	✓	✓	✓			
		TEM-1, TEM-2, SHV-1	✓	✓				✓	✓	✓	✓	✓	✓	✓
		CTX-M-15, GES-1, VEB-1	✓	✓	✓		✓	✓	✓	✓	✓	✓	✓	✓
		IRT, SHV-10, TEM-30	✓					✓	✓	✓	✓	✓	✓	✓
		CARB-1, PSE-1	✓					✓	✓	✓	✓			
		KPC, SME-1, GES-2	✓	✓	✓	✓	✓	✓	✓	✓	✓	✓	✓	✓
	C	AmpC, P99, ACT-1, MIR-1		✓				✓	✓	✓	✓	✓	✓	✓	
		GC1, CMY-37		✓						✓	✓	✓		
	D	OXA-1, OXA-10	✓					+/−	+/−		✓	✓		✓
		OXA-11, OXA-15	✓		✓		✓	+/−	+/−		✓			
		OXA-23, OXA48	✓			✓		✓	+/−		✓	+/−	✓	✓
MBL	B	IMP, VIM, NDM	✓	✓	✓	✓						✓	✓	✓
		CphA, Sfh-1				✓								✓

Abbreviations: BL, β-lactamase; MBL, metallo-β-lactamase; Pen, penicillins; Cep, cephalosporins; ECep, extended-spectrum cephalosporins; Cbn, carbapenems; Mb, monobactams. Symbols: ✓, substrate or inhibitor; +/−, variable activity.

**Table 2:** Main pharmacokinetic characteristics of BLIs and their beta-lactam companion for comparison.

Drug	CL ^1^ (L/h)	V_d_ (L)	t_1/2_ (h)	PPB (%)	References
AVI	1.59	18.0	2.0	8	[45]
CAZ	1.54	22.0	2.0	10	[45]
AZT	140	29.4	1.7	77	[46,47]
CEF	1.8–3.0	28.3	2.5	20	[48]
VAB	10.5	19.0	2.25	33	[34,49,50]
MER	7.7	21.0	2.30	2	[34,49,50]
REL	8.1	21	1.7	22	[34,51]
IMI	8.4	21.7	1.1	20	[35,51,52]
DUR	10.3	31.6	2.5	-	[52]
SUL	2.4	12.0	1.8	38	[52,53]
ZID	7.4	17.4	1.9	<15	[54]
NAC	8.8	20.6	2.4	-	[55]
TAN	5.8	30–50	6.5	-	[56,57]

^1^ Abbreviations: ATM, aztreonam; AVI, avibactam; AZT, aztreonam; CAZ, ceftazidime; CEF, ceftaroline fosamil; DUR, durlobactam; IMI, imipenem; MER, meropenem; NAC, nacubactam; REL, relebactam; SUL, sulbactam; TAN; taniborbactam; VAB, vaborbactam; ZID, zidebactam; CL, clearance; V_d_, volume of distribution; t_1/2_, terminal elimination half-life; PPB, plasma protein binding.

**Table 3:** β-lactam plus BLI combinations registered for clinical use in Europe or in clinical evaluation for the treatment of several infections.

Drugs and Dosage	Clinical Use	Therapeutic Indications (Duration of Treatment)	Pediatric Use	Ref.
CAZ/AVI ^1^ 2/0.5 g q8h 2-h IV infusion	YES	cIAI (5–14 days) cUTI (5–10 days) Pyelonephritis (5–10 days) HAP (7–14 days) VAP (7–14 days) Aerobic G- infections (variable)	YES (age ≥3 months)	[45]
MER/VAB 2/2 g q8h 3-h IV infusion	YES	cIAI (5–10 days) cUTI (5–10 days) Pyelonephritis (5–10 days) HAP (7–14 days) VAP (7–14 days) Aerobic G- infections (variable) Bacteremia (variable)	NO	[50]
REL/IMI/CIL 0.25/0.5/0.5 g q6h 0.5-h IV infusion	YES	HAP (7–14 days) VAP (7–14 days) Infections from Aerobic G- (variable) Bacteremia (variable)	NO	[51]
REL/IMI 0.25/0.5 g q6h 0.5-h IV infusion	NO	HAP (variable) VAP (variable) cUTI (variable) cIAI (variable)	-	[100,101,102]
DUR/SUL	NO	Phase III studies: cUTI, HAP, VAP	-	[103,104]
DUR/IMI/CIL	NO	Studies in HV	-	[61]
ZID/CEF	NO	Phase III studies: cUTI, HAP, VAP	-	[104]
NAC/CEF or AZT	NO	Phase I studies: cUTI	-	[104]
TAN/CEF	NO	Phase III studies: cUTI	-	[104]

^1^ Abbreviations: AVI, avibactam; AZT, aztreonam; CAZ, ceftazidime; CEF, cefepime; DUR, durlobactam; IMI, imipenem; IMI / CIL, imipenem/cilastatin; MER, meropenem; REL, relebactam; SUL, sulbactam; VAB, vaborbactam; cIAI, complicated intra-abdominal infection; cUTI, complicated urinary tract infection; HAP, hospital-acquired pneumonia; VAP, ventilator-associated pneumonia; HV, healthy volunteers; IV; intravenous.
